# Effects of *CYP3A5* Genotype on Tacrolimus Pharmacokinetics and Graft-versus-Host Disease Incidence in Allogeneic Hematopoietic Stem Cell Transplantation

**DOI:** 10.3390/ph17050553

**Published:** 2024-04-25

**Authors:** Daniel N. Marco, Mònica Molina, Ana-María Guio, Judit Julian, Virginia Fortuna, Virginia-Lucila Fabregat-Zaragoza, María-Queralt Salas, Inés Monge-Escartín, Gisela Riu-Viladoms, Esther Carcelero, Joan Ramón Roma, Noemí Llobet, Jordi Arcarons, María Suárez-Lledó, Laura Rosiñol, Francesc Fernández-Avilés, Montserrat Rovira, Mercè Brunet, Carmen Martínez

**Affiliations:** 1 Hematopoietic Stem Cell Transplantation Unit, Hematology Department, Institute of Cancer and Hematological Diseases, Instituto de Investigación Biomédica August Pi i Sunyer (IDIBAPS), Hospital Clínic, 08036 Barcelona, Spain; dnmarco@clinic.cat (D.N.M.); mmolinam@clinic.cat (M.M.); amguio@clinic.cat (A.-M.G.); mqsalas@clinic.cat (M.-Q.S.); nllobet@clinic.cat (N.L.); jarcarons@clinic.cat (J.A.); msuarezl@clinic.cat (M.S.-L.); lrosinol@clinic.cat (L.R.); ffernand@clinic.cat (F.F.-A.); mrovira@clinic.cat (M.R.); 2 Pharmacology and Toxicology Laboratory, Biochemistry and Molecular Genetics Department, Biomedical Diagnostic Center, IDIBAPS, CIBERehd, Hospital Clínic, 08036 Barcelona, Spain; jjpena@clinic.cat (J.J.); vfortuna@clinic.cat (V.F.); mbrunet@clinic.cat (M.B.); 3 Department of Immunology, Hospital Clínic, 08036 Barcelona, Spain; vfabrega@clinic.cat; 4 Department of Pharmacy, Pharmacy Service, Hospital Clínic, 08036 Barcelona, Spain; monge@clinic.cat (I.M.-E.); griu@clinic.cat (G.R.-V.); ecarcele@clinic.cat (E.C.); roma@clinic.cat (J.R.R.)

**Keywords:** tacrolimus, therapeutic drug monitoring, *CYP3A5* genotype, graft-versus-host disease, preemptive pharmacogenetics, allogeneic hematopoietic stem cell transplantation

## Abstract

Tacrolimus (Tac) is pivotal in preventing acute graft-versus-host disease (GVHD) after allogeneic hematopoietic stem cell transplantation (alloHSCT). It has been reported that genetic factors, including CYP3A5*3 and CYP3A4*22 polymorphisms, have an impact on Tac metabolism, dose requirement, and response to Tac. There is limited information regarding this topic in alloHSCT. The *CYP3A5* genotype and a low Tac trough concentration/dose ratio (Tac C_0_/D ratio) can be used to identify fast metabolizers and predict the required Tac dose to achieve target concentrations earlier. We examined 62 Caucasian alloHSCT recipients with a fast metabolizer phenotype (C_0_/dose ratio ≤ 1.5 ng/mL/mg), assessing *CYP3A5* genotypes and acute GVHD incidence. Forty-nine patients (79%) were poor metabolizers (2 copies of the variant *3 allele) and 13 (21%) were CYP3A5 expressers (CYP3A5*1/*1 or CYP3A5*1/*3 genotypes). CYP3A5 expressers had lower C_0_ at 48 h (3.7 vs. 6.2 ng/mL, *p* = 0.03) and at 7 days (8.6 vs. 11.4 ng/mL, *p* = 0.04) after Tac initiation, tended to take longer to reach Tac therapeutic range (11.8 vs. 8.9 days, *p* = 0.16), and had higher incidence of both global (92.3% vs. 38.8%, *p* < 0.001) and grade II-IV acute GVHD (61.5% vs. 24.5%, *p* = 0.008). These results support the adoption of preemptive pharmacogenetic testing to better predict individual Tac initial dose, helping to achieve the therapeutic range and reducing the risk of acute GVHD earlier.

## 1. Introduction

Allogeneic hematopoietic stem cell transplantation (alloHSCT) is a well-established treatment for hematologic diseases that cannot be cured with conventional therapies. The most common life-threatening complication after transplantation is graft-versus-host disease (GVHD), which occurs when immunocompetent T cells from the donor (the graft) recognize the recipient tissues (the host) as foreign. One of the cornerstones of transplant success is adequate GVHD prophylaxis [1,2]. Tacrolimus (Tac) is the main component of many current immunosuppressive regimens for the prevention of GVHD following alloHSCT [1,3]. However, Tac dosing in the setting of alloHSCT is complex due to its narrow therapeutic index and high interpatient and intrapatient pharmacokinetic variability. Excessive variations in Tac blood concentration have been associated with poor clinical outcomes in terms of GVHD incidence (underdosing) and toxicity (overdosing) [3,4,5].

In current clinical practice, Tac is usually initiated at a fixed dose based on patient weight and then titrated using therapeutic drug monitoring (TDM) employing Tac blood trough concentration (C_0_), which is usually maintained in the range of 5–15 ng/mL [2,3,4]. Unlike in solid organ transplantation (SOT), in alloHSCT Tac is maintained for the first 3–6 months post-transplant and then tapered and discontinued in the absence of GVHD by the 6th–12th month post-transplant [6]. Therefore, exposure to Tac during the immediate period after the HSCT period is of utmost importance to prevent subsequent GVHD [4,7,8]. Furthermore, subtherapeutic Tac trough concentrations (C_0_ < 5 ng/mL) at 48 h or 7 days after transplant has been associated with an increased incidence of acute GVHD (GVHD) [9,10]. 

During the last decade, in the context of SOT, it has been demonstrated that pharmacogenetic biomarkers influencing Tac exposure and response have the potential to enable individualization of the starting dose and favor the achievement of Tac target concentrations first days after transplantation [11,12,13]. Genetic factors including CYP3A5*3, CYP3A4*22, CYP4A4*1B, POR*28, and *ABCB1* genetic variants have been reported frequently for their influence on Tac dose requirements [13,14,15]. Based on the available data, the effect of CYP4A4*1B, POR*28, and *ABCB1* on Tac exposure seems to be much less clinically relevant as determinant biomarkers than the CYP3A5*3 or the cluster CYP3A5*3/CYP3A4*22 genotypes, at least in the Caucasian population [16].

A single nucleotide polymorphism in the *CYP3A5* gene involving an A to G transition at position 6986 within intron 3 (rs776746) is strongly associated with CYP3A5 protein expression. Thus, the CYP3A5*3 allele causes a cryptic splice site resulting in protein truncation and a non-functional CYP3A5 protein [17]. Patients carrying at least one CYP3A5*1 allele (the wild type allele) are considered CYP3A5 expressers and this genotype may predict the metabolic phenotypes of extensive metabolizers (CYP3A5*1/*1) or intermediate metabolizers (CYP3A5*1/*1). Whereas those homozygous for the CYP3A5*3 (CYP3A5*3/*3) are non-CYP3A5 expressers and considered poor metabolizers, with a frequency around 80% in Caucasians [11,13]. Individual CYP3A5 expressers require almost double the conventional starting Tac dose to achieve target concentrations [11].

The CYP3A4*22 SNP (rs35599367) is another polymorphism that has been investigated in relation to Tac pharmacokinetics. This polymorphism is located 192 bp upstream of exon 7, the substitution C>T in this allele alters RNA splicing resulting in both decreased CYP3A4 mRNA expression (mainly in the liver) and decreased enzymatic activity. This SNP has an allele frequency of around 6% in Caucasians [18].

Other genetic variants such as the CYP3A*1B (rs2740574), and the CYP-mediated drug oxidation POR*28 (rs1057868) have been investigated. CYP3A4*1B was linked to an increased CYP3A4 activity; however, this has not been a consistent finding. Some groups have reported a higher Tac dose requirement of patients carrying the variant allele but this is largely explained by the strong linkage with the CYP3A5*1 allele [19].

Taking into account only the *CYP3A5* genotype, patients who are CYP3A5 expressers (CYP3A5*1/*3, CYP3A5*1/*1) may be classified as extensive metabolizers when their genotype corresponds to CYP3A5*1/*1 and intermediate metabolizers for genotype CYP3A5*1/*3 [11]. As a result, it has been observed in kidney transplant recipients, that Tac oral clearance is about 2.4-fold higher in CYP3A5 expressers compared with non-expressers, leading to 2.5-fold higher dose requirements in CYP3A5 expressers [20]. Considering that CYP3A5 expressers require almost double the conventional Tac dose to achieve target concentrations, these patients are more susceptible to presenting a low C_0_/dose ratio. In solid organ transplantation (particularly in kidney transplantation), a low C_0_/dose ratio (inferior of 1.05 ng/mL/mg) has been associated with worse graft and patient clinical evolution [21,22]. 

Pharmacogenetic testing of CYP3A5*3 and CYP3A4*22 before transplant could play a role in allowing individualization of the starting dose of Tac to achieve target concentrations earlier. This concept could be referred to as “preemptive pharmacogenetics” [23]. There are few studies about the impact of CYP3A5*3 polymorphisms on alloHSCT outcomes, mainly conducted in the Asiatic population [24,25,26,27]. It has been suggested that the expression of CYP3A5 and a low C_0_/dose ratio seem to be similar or interchangeable factors by which to identify fast metabolizers and predict the Tac dose required to achieve target concentrations [21]. However, in the post-transplant period, other variables may also play a role: patient weight, age, hematocrit, physiopathology, and concomitant medications (drug–drug interactions) such as corticosteroids and azoles [20,23]. Standardization of antifungal prophylaxis with azoles may have a confounding effect regarding patient *CYP3A5* genotype, as azoles are known to be potent inhibitors of CYP3A [28]. Similarly, corticosteroids are known to induce CYP3A enzyme activity [29] and may have the opposite clinical confounding factor. Taken together, these factors may confound the net genetic effect and its influence on Tac dose requirement. 

The aim of the study was to assess the potential impact of *CYP3A5* and *CYP3A4* genotypes on acute GVHD incidence in alloHSCT transplant recipients. To carry out this project, we selected a group of Caucasian alloHSCT recipients with a fast metabolizer phenotype. We have identified as fast metabolizer phenotype those patients with a C_0_/dose ratio ≤ 1.5 ng/mL/mg, considering that, in our cohort, CYP3A5 non-expressers show a C_0_/dose ratio >1.5 ng/mL/mg. 

## 2. Results

### 2.1. Baseline Characteristics of the Patients

Sixty-two patients were included in the study. Patient and transplant characteristics are summarized in Table 1. The mean age was 49 years (range 18–69 years, SD 15 years), and 52% were male. The most common primary diagnosis was acute leukemia (42%) or myelodysplastic syndrome (22%), followed by other malignant hematologic diseases. Thirty-five (56%) patients underwent RIC alloHSCT and the majority of patients had achieved either complete (58%) or partial (16%) remission at the time of alloHSCT. More than half of the patients (56%) had an HLA-matched donor, mostly from unrelated donors. Most of the patients received oral Tac formulations (73% QD, 24% BID), and 77% received PTCy-based for GVHD prophylaxis. 

### 2.2. CYP3A5 and CYP3A4 Genotypes and Tac Pharmacokinetics

Forty-nine patients (79%) were identified as poor metabolizers (CYP3A5 non-expressers) whereas thirteen (21%) patients were identified as CYP3A5 expressers, twelve were intermediate metabolizers (CYP3A5*1/*3 genotype) and one was extensive metabolizer (CYP3A5*1/*1 genotype). The minor allele frequency (MAF) of CYP3A5*1 was 11% and the MAF of CYP3A4*22 was 2%. The data were close to the MAF of CYP3A5*1 (11%) and CYP3A4*22 (4%) in the dbSNP of the Alfa database (https://www.ncbi.nlm.nih.gov/snp/docs/gsr/alfa/ (accessed on 15 Aril 2024)). 

Both groups were comparable in all transplant-related and patient characteristics except for the use of PTCy as part of a GVHD prophylaxis regimen, with a preponderance of PTCy-based regimens in intermediate and extensive metabolizers (100% vs. 71%, *p* = 0.03). Clinical variables that affect Tac pharmacokinetics, such as patient weight, hematocrit, and the use of azoles and corticosteroids, were similar in both groups.

**Figure 1:**
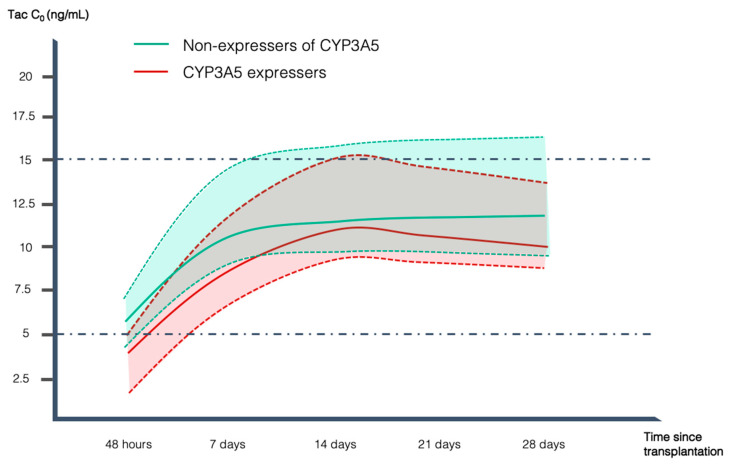
Diagram displaying the median Tac trough concentration (C_0_) along the study period for both poor metabolizers (non-expressers of CYP3A5) (green) and intermediate and extensive metabolizers (CYP3A5 expressers) (red). Continuous lines represent the median C_0_ values whereas discontinuous lines represent p25 and p75, respectively. Discontinuous dotted line signals desired therapeutic range.

Intermediate and extensive metabolizers had lower C_0_ at TISS (3.7 vs. 6.2 ng/mL, *p* = 0.03) and at 7 days (8.6 vs. 11.4 ng/mL, *p* = 0.04) after Tac initiation compared to poor metabolizers and tended to take longer to reach the therapeutic range of 5–15 ng/mL (11.8 vs. 8.9 days, *p* = 0.16) (Figure 1). Specifically, only 23% of intermediate and extensive metabolizers achieved therapeutic range at TISS compared to 59% of poor metabolizers (*p* = 0.029). In addition, the group of intermediate and extensive metabolizers had a higher proportion of patients with C_0_ < 5 ng/mL at TISS (77% vs. 39%, *p* = 0.026) and at 7 days (23% vs. 2%, *p* = 0.03). No significant differences were observed between poor and intermediate and extensive metabolizers in Tac C_0_ concentrations at 14 days, 21 days, and 28 days measurements (Figure 1). 

Whereas in poor metabolizers the mean Tac dose was stable from 48 h to 7 days (11.6 vs. 11.3 mg, *p* = 0.7), intermediate and extensive metabolizers had a mean dose increase of 3.6 mg (10 vs. 13.6 mg, *p* = 0.025). Accordingly, the C_0_/dose ratio at 48h (0.38 vs. 0.76, *p* = 0.16) and at 7 days (0.69 vs. 1.04, *p* = 0.038) were lower in intermediate and extensive metabolizers whereas there were no differences in the mean monthly C_0_/dose ratio (0.98 vs. 1.08, *p* = 0.28) or at 14 days, 21 days, and 28 days measurements.

Regarding the *CYP3A4* genotype, two patients were identified as heterozygous carriers of CYP3A4*22 (CYP3A4*1/22). One of them was a concomitant expresser of CYP3A5 (CYP3A5*1/*3) and the other was a non-expresser of CYP3A5 (CYP3A5*3/*3). Any of them reached the desired Tac C_0_ until day 5 after Tac initiation. Whereas the first patient (extensive metabolizer) maintained a relatively stable Tac dose throughout the study period, the second patient (poor metabolizer) presented supratherapeutic concentrations at day 21 days that led to a temporary suspension of Tac administration.

### 2.3. CYP3A5 and CYP3A4 Genotypes and Acute GVHD Incidence

**Figure 2:**
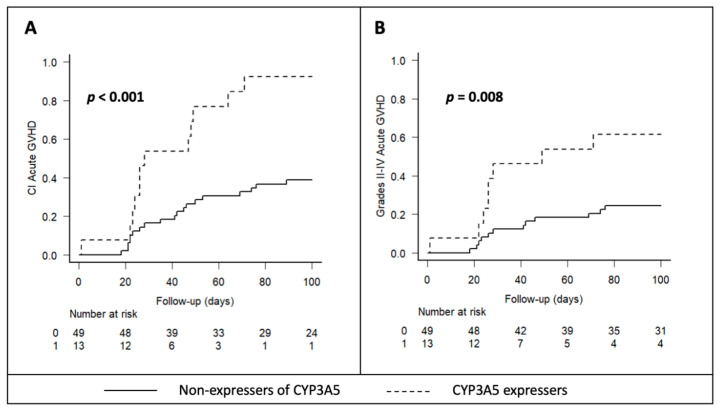
Kaplan–Meyer analyses of the effects of *CYP3A5* expression on the cumulative rates of GVHD (**A**) and clinically relevant (grades II–IV) GVHD (**B**).

During the study period, 52% (32 patients) developed acute GVHD (grades I–IV), with a 100-day cumulative incidence of 50% (95% CI 37–62%). Out of these, 63% (20 patients) presented with clinically relevant acute GVHD (grades II-IV), with a cumulative incidence of 33% (95% CI 17–47%). Compared to poor metabolizers, intermediate and extensive metabolizers had higher incidence of both global acute GVHD (92.3% [25–52%] vs. 38.8% [95% CI 36–99%], *p* < 0.001) and grade II–IV acute GVHD (61.5% [95% CI 28–83%] vs. 24.5% [95% CI 14–37], *p* = 0.008) (Figure 2). In fact, only one extensive metabolizer patient did not develop acute GVHD during the study period. In the univariate analysis, the other variable associated with a higher incidence of acute GVHD was a lower hematocrit (referred to as <p50) at 48 h post-transplant (67% vs. 35%, *p* = 0.02). The remaining variables included in the univariate and multivariate analysis for clinically relevant acute GVHD incidence are shown in Table 2. Multivariate analysis confirmed CYP3A5 intermediate and extensive metabolizers as an independent risk factor for acute GVHD (HR 3.5 [95% CI 1.5–8.3], *p* = 0.003) as well as clinically relevant acute GVHD (grades II-IV) (HR 4.5 [95% CI 2.4–8.6], *p* < 0.001). 

Both patients previously identified as CYP3A4*22 carriers presented grade II acute GVHD.

### 2.4. CYP3A5 and CYP3A4 Genotypes and Tac-Related Adverse Events

Forty-four (71%) patients presented at least one AKI episode during the study period. Of those, 25 (57%) were identified as moderate to severe AKI (KDIGO grades 2 and 3). The incidence of any grade of AKI was similar between intermediate and extensive and poor Tac metabolizers. However, intermediate and extensive metabolizers showed a trend toward a higher incidence of moderate to severe AKI (62% vs. 35%, *p* = 0.11) compared to poor metabolizers, and this trend was also present with respect to the incidence of severe AKI (KDIGO grade 3) (23% vs. 8%, *p* = 0.15). Similarly, both the mCr (1.94 vs. 1.4 mg/dL, *p* = 0.01) and the mCr/bCr ratio were higher among intermediate and extensive metabolizers (2.4 vs. 1.9, *p* = 0.11) than in the poor metabolizer group. Supratherapeutic levels of Tac C_0_ (>15 ng/mL) at different time points after transplant were not significantly associated with AKI incidence or severity. There were no differences in neurotoxicity, graft failure, and TMA incidence between both groups.

Both patients previously identified as CYP3A4*22 carriers presented a grade 2 AKI episode during the study period.

## 3. Discussion

In our study, the obtained results demonstrate that CYP3A5*1/*3 and CYP3A5*1/*1 genotypes were significantly associated with infra-therapeutic Tac concentrations at 48 h and 7 days after Tac initiation, took longer to reach the therapeutic range, and had a higher risk of acute GVHD and renal toxicity. 

In our cohort, which exclusively constituted Caucasian alloHSCT recipients, there was a solid association between CYP3A5 intermediate and extensive metabolizers (CYP3A5*1/*3 and CYP3A5*1/*1 genotypes) and lower concentrations of Tac in the first measurements (48 h and 7 days) coupled with lower rates of achievement of the lower limit of therapeutic range. The mean delay to achieve a therapeutic range for intermediate and extensive metabolizers was 2.9 days with respect to poor metabolizers. Consistent with the previous findings, intermediate and extensive metabolizers had a lower C_0_/dose ratio at 48 h and 7 days of treatment. Regarding Tac-related outcomes, CYP3A5*1/*3 and CYP3A5*1/*1 genotypes were an independent risk factor for a higher incidence of clinically significant acute GVHD grade II-IV (OR 6.4, *p* = 0.009) in our cohort. This association remained significant regardless of lower hematocrit, which also was associated with a higher rate of acute GVHD. This latter fact has been previously reported and is explained because erythrocytes act as a reservoir of Tac in the organism [30,31]. Despite not achieving statistical significance, there was a trend to higher incidence of clinically relevant episodes of AKI (KDIGO 2–3) and higher worsening of serum creatinine levels after transplantation in intermediate and extensive metabolizers. This fact could be in line with the hypothesis that patients with faster metabolizer phenotypes typically receive higher doses of Tac (lower C_0_/D ratios) in order to achieve the desired therapeutic range. Thus, these patients are likely to be exposed to higher Tac peak concentrations and this may explain an increased risk of AKI and other Tac-related adverse events [22,32]. On the other hand, lower nephrotoxicity is expected in patients exposed to lower Tac concentrations.

These findings indicate that achieving Tac treatment goals is more difficult in intermediate and extensive Tac metabolizers (CYP3A5 expressers), as higher Tac doses are required, resulting in lower C0/Dose ratios. These pharmacokinetic differences were observed at 48 h as well as at 7-day measurements after Tac initiation. These data could have relevant clinical implications after alloHSCT. Lower Tac C_0_ within the early period after alloHSCT has been associated with a higher incidence of acute GVHD [9,10] despite therapeutic drug monitoring and dose adjustment. Accordingly, we have observed a higher rate of clinically significant acute GVHD and AKI in intermediate and extensive Tac metabolizers patients. These results are consistent with the findings of Khaled et al., who reported higher concentrations of Tac in patients with the CYP3A5*3/*3 genotype [24]. Onizuka et al. also reported higher Tac C_0_ and C_0_/doses in patients with CYP3A5*3/*3 genotype compared to those with CYP3A5*1/*1 and CYP3A5*1/*3 genotypes [25]. However, Yamashita [26] et al. and Hamadeh et al. [27] did not find any differences in pharmacokinetic variables between patients with CYP3A5*3/*3 genotype and those with CYP3A5*1/*1 and CYP3A5*1/*3 genotypes. Remarkably, the use of fungal prophylaxis regimens and corticosteroid use differs among the aforementioned studies. In view of the fact that alloHSCT patients usually require several medications at the same time to prevent both transplant-related complications and infectious diseases, the isolation of the net effect of the genetic role of Tac metabolism is complex. Nevertheless, due to the use of standardized conditioning and GVHD prophylaxis regimens in our center, the first determination of Tac (TISS) would be a reasonably accurate point of comparison between patients and our results show relevant differences attributable to genetic differences in this early phase of post-transplant period. The incidence of acute GVHD and its association with the *CYP3A5* genotype has only been explored in the studies by Khaled and Yamashita, which also identified CYP3A5*1/*1 as an independent risk factor for acute GVHD. 

In the studied population, only 21% of patients with the fast metabolizer phenotype (C_0_/D ratio < 1.5 ng/mL/mg) were CYP3A5 expressers (intermediate and extensive metabolizers). This suggests that additional factors such as drug–drug interactions, mainly the potent induction of Tac metabolism by corticosteroids, could notably influence the pharmacokinetics of Tac. Among the patients non-expressers of CYP3A5, 45% were receiving corticosteroids during the post-transplant period. 

The study’s main limitation is the relatively small number of patients included. This may have underpowered some significant differences in the measured variables in the cohort, particularly patient and transplant characteristics known to be associated with increased acute GVHD. There are few data published in the field of alloHSCT focusing on the Caucasian population, but our cohort displays the expected genotype distribution in this ethnic population (19% of patients CYP3A5*1/*3, 2% CYP3A5*1/*1, 3% CYP3A4*1/*22) [33,34]. Furthermore, the limited number of patients with the CYP3A4*22 allele prevents drawing definitive conclusions and requires validation in future studies. In our cohort of alloHSCT patients, all individuals were treated with azoles, which are potent inhibitors of CYP3A, and 50% of them received corticosteroids, which are potent inducers of CYP3A, at some point during treatment. The distribution of azoles and corticosteroid use was comparable between the two *CYP3A5* genotype groups. The *CYP3A5* genotype appears to have overshadowed the potential phenoconversion effect of those medications, according to our results. During the early period after transplant, Tac concentrations were found to be more influenced by pharmacogenetic factors than drug–drug interactions.

Given the strong association found in this study between CYP3A5*1/*3 and CYP3A5*1/*1 with Tac pharmacokinetics in the immediate post-transplant period and acute GVHD incidence, these results support the adoption of preemptive pharmacogenetic testing as a tool to better predict each patient’s Tac initial dose, helping to achieve the therapeutic range and reduce the risk of acute GVHD earlier.

## 4. Materials and Methods

### 4.1. Patients and Donors

The present study was performed at the Hospital Clínic in Barcelona (Spain) between February 2015 and December 2019. The study population consisted of alloHSCT Caucasian recipients with a fast metabolizer phenotype defined by a Tac C_0_/Dose ratio ≤ 1.5 ng/mL/mg. Eligibility criteria for transplant were as follows: age 18 to 69 years, first alloHSCT for malignant hematological disease, an Eastern Cooperative Oncology Group performance status ≤ 2, left ventricular ejection fraction ≥ 35%, forced expiratory volume in 1 s and forced vital capacity ≥ 40% of predicted, and adequate hepatic function (total bilirubin ≤ 3.0 mg/dL or absence of clinically significant liver disease). Only those patients with available information on Tac trough concentrations were considered eligible for the study. The protocol received institutional review board approval, and all participants provided signed informed consent.

### 4.2. Treatment Protocol and Supportive Care

Specific conditioning regimens used were based on the type of hematological disease and patient characteristics in accordance with institutional protocols. Patients aged > 50 years or previously submitted to an autologous HSCT received a reduced-intensity conditioning (RIC) regimen instead of myeloablative conditioning (MAC). All patients received peripheral blood as stem cell source.

Tac was initiated using either the intravenous formulation (0.03 mg/kg as a 24 h perfusion), the oral twice-daily [BID: bis in die] formulation (Tacni^®^, 0.06 mg/kg), or the one-daily modified-release [QD: quaque die] formulation (Advagraf^®^, 0.12 mg/kg) from day −1 or day +5 in those patients receiving post-transplant high-dose cyclophosphamide (PTCy). Tac was continued therapeutic until day +90 and then, tapered if acute GVHD grade II-IV was absent. 

Patients that received GVHD prophylaxis regimen based on high-dose PTCy received 50 mg/kg IV once daily on days +3 and +4, along with Mesna at 80% of the Cy dose (divided into three doses). No patient received ATG or alemtuzumab for GVHD prevention. Granulocyte colony-stimulating factor was routinely given in haploidentical alloHSCT starting on day +7 until the absolute neutrophil count reached 1000 cells/mm [3] for 3 consecutive days. 

Antimicrobial prophylaxis was administered according to our institutional practice guidelines. Standard prophylaxis included levofloxacin at a dose of 500 mg/24 h from day 0 until neutrophil engraftment, fluconazole at a dose of 400 mg c/24 h until day +60, and acyclovir at a dose of 400 mg c/12 h until day +365 (for patients who were seropositive for herpes simplex virus). Standard *Pneumocystis jirovecii* prophylaxis was used until CD4^+^ T cell recovery (>200 cells/μL) and/or until immunosuppression was discontinued. If any deviation in standard dose of fluconazole occurred, including suspension, increment, or a change by other azoles; Tac dose was adjusted or closely followed by whole blood concentration monitoring to adapt it to new circumstances and minimize under- or over-exposure. Cytomegalovirus (CMV) quantitative polymerase chain reaction (PCR) was performed weekly trough to at least day +60, and pre-emptive therapy was initiated if viral reactivation was detected (a PCR result above 1000 IU/mL or two consecutive rising values), according to standard practice recommendations.

### 4.3. Tac Pharmacokinetic Parameters

Tac whole blood C_0_ monitoring was routinely assessed three times per week from the day following the initiation of Tac until patient hospital discharge, and then, once weekly thereafter [35]. In the absence of acute GVHD or Tac-related toxicities, Tac weekly sampling was maintained usually during the first month after transplantation. We developed and validated ultra-high-performance liquid chromatography–tandem mass spectrometry (UPLC/MS/MS) procedure, under the European Medicines Agency (EMA) guideline [36]. The method was linear from 1 to 40 ng/mL of Tac. The lower limit of quantification is 1 ng/mL of Tac. Within and between-run accuracy and precision fulfilled the validation criteria. Tac doses were adjusted in order to achieve and maintain a Tac C_0_ between 5 and 15 ng/mL. This technique is subject to Tac Proficiency Testing from LGC Standards as an external quality control. 

The primary variables of interest for the present study were Tac target concentrations at initial steady state (TISS), C_0_ at 7, 14, 21, and 28 days after Tac initiation, and Tac C_0_/dose ratio. The Tac C_0_/dose ratio was calculated as a quotient and was given as a fraction of unity. TISS was defined as the first whole blood concentration at 48 h post-initiation of Tac, which is approximately 4–5 times the half-life of the drug (12 h). 

Successive hematocrit levels for each patient were recorded coinciding with C_0_ determinations at 48 h, 7, 14, 21, and 28 days after Tac initiation. 

### 4.4. CYP3A5 and CYP3A4 Genetic Polymorphism Analysis

Genomic DNA was obtained from blood samples of the recipients before the alloHSCT using MagNA Pure LC DNA Isolation Kit in an automatic DNA extractor (MagNa Pure^®^ System, Roche Applied Science, Indianapolis, Indiana) according to the manufacturer’s instructions. Allelic discrimination analysis was performed to determine the allelic status of CYP3A5*3 (rs776746) and CYP3A4*22 (rs35599367). CYP3A5*3 (C_26201809_30 probe) and CYP3A4*22 (C_59013445_10 probe) polymorphisms were genotyped by quantitative PCR (qPCR), in a QuantStudio 5.0 instrument (Applied Biosystem, Foster City, CA, USA). PCR mixture consisted of a 2.5 µL TaqMan Universal PCR mastermix, 0.25 µL TaqMan Drug Metabolism Genotyping Assay mix (20×), and 2.25 µL of DNA [20 ng] completed to 50 µL with nuclease-free water. The PCR run method was as follows: an initial step at 95 °C for 10 min, followed by 40 cycles: step 1 at 95 °C for 15 s and step 2 at 60 °C for 60 s. This technique is subject to external quality control by Referenzinstitut für Bioanalytik (RfB).

According to Clinical Pharmacogenetics Implementation Consortium [11], for the purpose of this study, and to focus only on *CYP3A5* genotype: patients with CYP3A5 expression were considered extensive metabolizers (CYP3A5*1/*1) or intermediate metabolizers (CYP3A5*1/*3) whereas patients with 2 copies of the variant *3 allele were considered poor metabolizers. In addition, patient carriers of CYP3A4*22 allele and CYP3A5 non-expressers were considered poor metabolizers. Whereas CYP3A4*22 noncarriers and CYP3A5 non-expressers (that account for about 80% of Caucasian population) were considered intermediate metabolizers [13]. 

### 4.5. Endpoints of the Study

The main objective of the study was to analyze the influence of *CYP3A5* and *CYP3A4* genotypes in Tac initial dose election to better prevent GVHD in a selected population of alloHSCT patients with fast metabolizer phenotype.

Secondary endpoints were the incidence of Tac-related toxicity events: acute kidney injury [AKI], neurotoxicity, and thrombotic microangiopathy [TMA]) according to *CYP3A5* and *CYP3A4* genetic polymorphisms. Diagnosis of acute GVHD was based on clinical and histopathological findings of affected organs and graded from I to IV according to MAGIC criteria [37]. AKI was defined according to the 2012 Kidney Disease: Improving Global Outcomes (KDIGO) Clinical Practice Guideline Criteria [38]. Basal (bCr) and maximum (mCr) post-alloHSCT serum creatinine levels were recorded in order to calculate AKI values (mCr/bCr ratio). The diagnosis and severity of TMA were determined following current recommendations [39,40]. Neurotoxicity severity was evaluated according to the National Cancer Institute Common Terminology Criteria for Adverse Events (AEs) (NCI CTCAE, version 4.0).

### 4.6. Statistical Analysis

Descriptive variables were presented using counts and percentages, and continuous variables were reported using mean and SD. The cumulative incidence of acute GVHD was calculated using cumulative incidence methods and accounting for death and relapse as competing events. Univariate and multivariate regression tests explored the impact of the primary variable of interest (*CYP3A5* genotype) on cumulative incidence of GVHD. Other variables that could potentially affect the incidence of acute GVHD were included in the statistical analysis: age at transplant, sex (male vs. female), disease status at transplant (chemosensitive disease [complete response/partial response] vs. stable disease/progression), CMV donor/recipient status, HLA compatibility (match related or unrelated donors vs. mismatched unrelated or haploidentical donor), donor to recipient relation sex (female to male vs. other), and conditioning regimen (MAC vs. RIC). The multivariate regression models included those variables found to be significant in the univariate analysis or clinically relevant. All *p*-values were 2-sided, and a *p*-value of <0.05 was considered to indicate statistical significance. All statistical calculations were performed using SPSS Statistics version 25 (SPSS INC, Chicago, IL, USA) and EZR software version 1.61.

## Figures and Tables

**Table 1:** Characteristics of patients and alloHSCT procedure.

Characteristics	All Patients N = 62	CYP3A5*3/*3 N = 49	CYP3A5*1 ^†^ N = 13	*p* Value ^††^
Mean age, years (SD)	49 (15)	49 (14)	50 (17)	0.7
Female sex	30 (48)	24 (49)	6 (46)	1
Mean Weight, kg (SD)	68.6 (12)	69.1 (12)	67.6 (14)	0.7
Primary diagnosis				
Acute leukemia	26 (42)	17 (35)	9 (69)	0.16
Myelodysplastic syndromes	14 (22)	12 (25)	2 (15)	0.3
Chronic myeloproliferative syndromes	11 (18)	10 (20)	1 (8)	-
Lymphoma/multiple myeloma	11 (18)	10 (20)	1 (8)	-
Disease status at transplant				
Complete response	36 (58)	27 (55)	9 (69)	0.6
Partial response	10 (16)	8 (16)	2 (15)	0.9
Stable disease/progression	16 (26)	14 (29)	2 (15)	0.4
Conditioning regimen				
Myeloablative	27 (44)	21 (43)	6 (46)	1
Reduced intensity	35 (56)	28 (57)	7 (54)	-
Donor type				
HLA match 10/10	35 (56)	28 (57)	7 (54)	1
Identical sibling	7 (11)	4 (8)	3 (23)	0.23
Haploidentical sibling	11 (16)	15 (31)	1 (8)	-
Discordant sex ^†††^	12 (19)	9 (18)	3 (23)	0.7
GVHD prophylaxis regimen				
PTCy-based	48 (77)	35 (71)	13 (100)	0.03
Tac IV	2 (3)	2 (4)	0 (0)	-
Tac BID	15 (24)	11 (22)	4 (31)	0.65
Tac QD	45 (73)	36 (74)	9 (69)	0.8
Hematocrit (SD)				
Day 0	0.29 (0.06)	0.29 (0.06)	0.29 (0.06)	0.9
At 48 h	0.29 (0.05)	0.29 (0.06)	0.29 (0.06)	0.9
Mean of first month	0.27 (0.03)	0.27 (0.03)	0.28 (0.03)	0.6
Change in azole regimen	15 (24)	11 (22)	4 (31)	0.72
Corticosteroids treatment	31 (50)	22 (45)	9 (69)	0.21

Values in brackets are presented in %. PTCy, post-transplant cyclophosphamide; BID, bis in die; IV, intravenous; QD, quaque die; SD, standard deviation. ^†^ Including either CYP3A5*1/*3 or CYP3A5*1/*1. ^††^ *p*-values have been omitted in boxes without enough number of patients. ^†††^ Male receptor and female donor.

**Table 2:** Univariate and multivariate analysis for II-IV GVHD incidence.

Characteristics		Univariate Analysis	*p*-Value	Multivariate Analysis, HR (95% CI)	*p*-Value
Patient age	Continuous variable (years)	48.5 vs. 49.7	0.75	1.01 (0.97–1.03)	0.7
Conditioning regimen	RIC (vs. myeloablative)	25.7 vs. 40.7	0.28	0.47 (0.2–1.09)	0.08
Disease status at transplant	Complete remission (vs. other)	31.4 vs. 33.3	1		
Donor sex	Female donor to male (vs. other)	41.7 vs. 30	0.5	0.81 (0.33–1.93)	0.64
Donor type	Any mismatch (vs. HLA matched)	33.3 vs. 31.3	1	1.78 (0.9–3.51)	0.1
Tac TISS	<5 ng/mL (vs. ≥5)	37.9 vs. 27.3	0.42		
Tac C_0_ at 7 days	<5 ng/mL (vs. ≥5)	75 vs. 29.3	0.09	7.3 (0.7–17.1)	0.1
Tac formulation	QD (vs. other)	31.1 vs. 35.3	0.7		
Hematocrit at 48 h	<25th percentile (vs. ≥25th)	56.3 vs. 23.9	0.029	5.3 (1.4–19.7)	0.01
	<50th percentile (vs. ≥50th)	39.4 vs. 24.1	0.28		
	<75th percentile (vs. ≥75th)	37.8 vs. 17.6	0.22		
CMV status	High risk (vs. other)	38.9 vs. 29.5	0.55	2.5 (0.3–13.1)	0.62
*CYP3A5*	*1/*3 or *1/*1 (vs. *3/*3)	61.5 vs. 24.5	0.019	4.51 (2.37–8.6)	<0.001

All values are displayed in % if not otherwise specified. HR, hazard ratio; RIC, reduced intensity conditioning; QD: quaque die; CMV, cytomegalovirus; Tac, tacrolimus; TISS, tacrolimus initial steady state; C_0_, tacrolimus trough concentration.

## Data Availability

All data are included within the article.

## Abbreviations

GVHD (graft-versus-host disease); alloHSCT (allogeneic hematopoietic stem cell transplantation); AKI (acute kidney injury); ATG (antithymocyte globulin); BID (bis in die); C_0_ (trough concentration); CI (confidence interval); CMV (cytomegalovirus); GVHD (graft-versus-host disease); IV (intravenous); MAC (myeloablative conditioning); PCR (polymerase chain reaction); PTCy (high-dose post-transplant cyclophosphamide); QD (quaque die); RIC (reduced intensity conditioning); SD (standard deviation); Tac (tacrolimus); TDM (therapeutic drug monitoring); TISS (tacrolimus initial steady state); TMA (thrombotic microangiopathy).
